# Concentric lamellae – novel microanatomical structures in the articular calcified cartilage of mice

**DOI:** 10.1038/s41598-019-47545-2

**Published:** 2019-08-01

**Authors:** Craig M. Keenan, Alison J. Beckett, Hazel Sutherland, Lakshminarayan R. Ranganath, Jonathan C. Jarvis, Ian A. Prior, James A. Gallagher

**Affiliations:** 1 Department of Musculoskeletal Biology, Institute of Ageing and Chronic Disease, University of Liverpool, William Henry Duncan Building, West Derby Street, Liverpool, L7 8TX UK; 2 Department of Cellular and Molecular Physiology, Institute of Translational Medicine, University of Liverpool, Nuffield Building, Crown Street, Liverpool, L69 3BX UK; 3 School of Sport and Exercise Sciences, Liverpool John Moores University, Tom Reilly Building, Byrom Street, Liverpool, L3 3AF UK; 4 Department of Clinical Biochemistry and Metabolism, Royal Liverpool University Hospital, Prescot Street, Liverpool, L7 8XP UK

**Keywords:** Microscopy, Bone

## Abstract

The structure, ultrastructure and function of hyaline articular cartilage (HAC) and subchondral bone (SCB), and their involvement in the pathogenesis of osteoarthritis (OA) have been extensively researched. However, much less attention has been focused on the intervening tissue, articular calcified cartilage (ACC) and its role in the initiation and progression of OA. Using both light microscopy (LM) and transmission electron microscopy (TEM), a study of ACC in wild type (WT) mice, and mice with genetic osteoarthropathies (AKU) was undertaken to further understand the role played by ACC in the early stages of OA.Tibio-femoral joints were obtained from BALB/c WT and BALB/c AKU mice aged between 7 and 69 weeks. One joint was processed for routine histological analysis. The tip of the medial femoral condyle (MFC), which contained HAC, ACC, and SCB, was dissected from the contra-lateral joint and processed for TEM.In WT and AKU mice novel microanatomical structures, designated concentric lamellae, were identified surrounding chondrocytes in the ACC. The lamellae appeared to be laid down in association with advancement of the tidemark indicating they may be formed during calcification of cartilage matrix. The lamellae were associated with hypertrophic chondrocytes throughout the ACC.Novel microanatomical structures, termed concentric lamellae, which were present around hypertrophic chondrocytes in the ACC are described for the first time. Their apparent association with mineralisation, advancement of the tidemark, and greater abundance in a model of osteoarthropathy indicate their formation could be important in the pathogenesis of OA and AKU.

## Introduction

The roles of hyaline articular cartilage (HAC) and subchondral bone (SCB) in the pathogenesis of osteoarthritis (OA) have been widely described, along with their structure, ultrastructure and function^1–4^. Much less attention has been focused on articular calcified cartilage (ACC)^5^ and its significance in the initiation and progression of OA has largely been ignored^6^. One possible explanation for this could be that the ultrastructure of ACC is notoriously difficult to study. Silberberg and colleagues performed TEM analysis on the femoral heads of mice of various ages but little attention was paid to ACC in these studies^7,8^. Recently, Hughes and colleagues used scanning electron microscopy (SEM) to describe in detail the orientation of chondrocytes and collagen fibers in the territorial and interterritorial matrices of murine HAC^9^. Similar to previous ultrastructural studies on mouse cartilage, there was far less detail on ACC than on HAC. Although the literature on the ultrastructure of ACC is scarce, and its role in the etiology of OA is not fully understood, it is known to play a significant role in the initiation and progression of the ultra-rare disease Alkaptonuria (AKU)^10,11^.

AKU is an ultra-rare autosomal recessive disorder characterized by elevated levels of homogentisic acid (HGA) in plasma. The HGA becomes deposited over the lifespan as a polymerized pigment in collagenous tissues, principally the cartilages of loaded joints, in a process known as ochronosis. In humans this results in an extreme and very severe OA phenotype in which cartilage is lost from the joints beyond the third and fourth decades of life. The pathogenesis of AKU has yet to be fully elucidated. However, Taylor and colleagues showed that pigment deposition in cartilage starts in the pericellular matrix (PCM) of chondrons, deep in the ACC and progresses up throughout HAC, leading to the early onset of the devastating osteoarthropathy associated with AKU^10^. It is generally accepted that OA initiates due to degradation of HAC, however it is clear from the work of Taylor and colleagues that ACC has more of a role than previously thought in the initiation and progression of osteoarthropathies^10^. Recently, there have been two murine models of AKU described which show pigmentation similar to that seen in the human condition^12,13^. In the latter of the two models, pigmentation was shown to be localized to chondrons in ACC confirming what Taylor *et al*. had identified in human tissue^10,12^. Until recently the only real, therapeutic option available for AKU patients was joint replacement surgery which is also the gold standard treatment for patients with OA. However, several pre-clinical and clinical studies have shown that a compound known as nitisinone is effective at preventing the build-up of HGA in plasma. Nitisinone was also shown to prevent pigment deposition in an animal model of AKU^14–17^.

This study was undertaken to provide a detailed analysis of the ultrastructure of all regions of articular cartilage, and to identify any differences between WT and AKU mice which could further the understanding of the development of the severe osteoarthropathy associated with AKU.

## Methods

### Mice

WT and AKU mice on a BALB/c background were used for all experiments. All work was carried out in accordance with the UK Home Office guidelines and regulations under the Animals (Scientific Procedures) Act 1986, and with approval from the University of Liverpool ethics committee. All mice were housed and maintained in the Biological Services Unit at the University of Liverpool, UK.

### Light microscopy

Tibio-femoral joints were harvested from WT and AKU mice aged between 7 and 69 weeks and fixed in 10% phosphate buffered formalin solution (PBFS). After 24 hours tissues were transferred to 12% EDTA to decalcify. Once decalcification was complete tissues were washed several times with PBS and processed for histological analysis using a Leica TP1020 processor (Leica, Germany). Following processing, tissues were embedded for coronal sectioning in paraffin wax. Tissue blocks were sectioned using a Leica RM2245 microtome (Leica, Germany), sections stained with H&E and Schmorl’s stain, and images captured using a Nikon Eclipse *Ci* microscope (Nikon, UK). Image analysis was performed using NIS Br elements software (Nikon, UK).

### Transmission electron microscopy

Following fixation in either 10% PBFS or 2.5% glutaraldehyde, the tip of the MFC, encompassing the HAC, ACC and SCB, was removed and post-fixed in 1% osmium tetroxide for 3 hours at RT, on bloc stained with 1% uranyl acetate for 24 hours at RT, dehydrated in ethanol and embedded in Agar 100 resin (Agar Scientific, UK). 70 nm sections were cut using a diamond knife (Diatome, Switzerland) on a Leica EM UC6 ultra-microtome (Leica, Germany). Sections were collected on formvar coated 100 mesh copper grids (TAAB, UK) and post-stained with uranyl acetate (5% by weight in 50% ethanol and 50% distilled water) followed by lead citrate. Grids were examined using a FEI 120 kV Tecnai G2 Spirit BioTWIN electron microscope, and all images captured with an SIS Megaview III camera.

### Ethical approval

All applicable international, national, and/or institutional guidelines for the care and use of animals were followed. This article does not contain any studies with human participants performed by any of the authors.

## Results

### Histological analysis of BALB/c AKU mice

**Figure 1:**
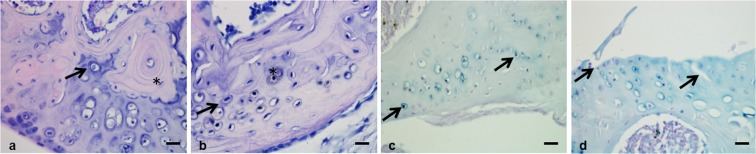
Histological examination of BALB/c AKU mice. (**a**) H&E staining of a 31 week old BALB/c AKU mouse showed the appearance of a concentric ring like structure around a chondrocyte in the articular calcified cartilage (ACC) of the medial femoral condyle (arrowed). Remodelling of the subchondral bone (SCB) was also seen which is an indication of osteoarthritis (OA) (*). (**b**) H&E staining of a 60 week old BALB/c AKU mouse also showed the appearance of concentric ring structures around a chondrocyte in ACC of the lateral femoral condyle (LFC) (arrowed), along with protrusion of the SCB into ACC. (**c**) Schmorl’s staining of a 60 week old BALB/c AKU mouse showed large numbers of pigmented chondrocytes, a hallmark of AKU, present throughout ACC of the LFC (arrowed). Pigmented chondrocytes were also visible in the H&E stained section (**b**) where they can be seen deep in ACC (*). (**d**) Analysis of a 49 week old BALB/c AKU mouse showed complete loss of the articular surface and vertical clefts running through the medial tibial plateau (arrowed), illustrating the severity of OA in these mice. Scale = 20 µm.

Sections from the tibiofemoral joints of AKU mice of varying ages were stained with H&E and Schmorl’s stain, and analysed using LM to determine if any hallmarks of OA, along with signs of ochronosis were present. At 31 weeks remodelling of the SCB was visible, along with what appeared to be concentric lamellar-like structures around a chondrocyte deep in ACC (Fig. 1a). Analysis of a 60 week old AKU mouse also showed a similar feature of concentric lamellae around a chondrocyte located along the SCB plate (Fig. 1b). Ochronotic pigmentation of chondrocytes and their surrounding matrices, located in ACC, was also visible (Fig. 1c). Hallmark signs of OA were observed in the mice, including loss of the articular surface and vertical clefts extending deep into the zones of HAC (Fig. 1d).

### Ultrastructural analysis of articular cartilage

**Figure 2:**
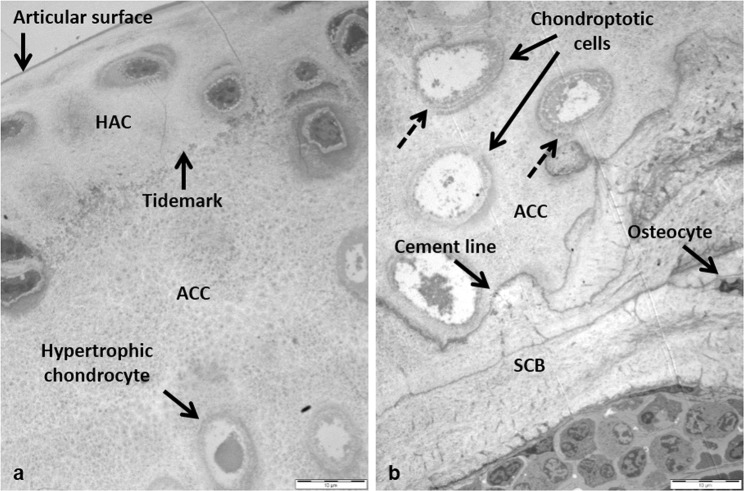
TEM micrographs of the medial femoral condyle from a 53 week old BALB/c AKU mouse. (**a**) HAC and ACC with the tidemark, which separates the two types of articular cartilage, have been labelled. A hypertrophic chondrocyte can be seen deep in the ACC. (**b**) Chondrocytes undergoing chondroptosis were visible in the ACC. Concentric lamellae were also visible surrounding the cells (dashed lines). The cement line which separates the ACC from the underlying SCB is highlighted (x1250). Tissue fixed in glutaraldehyde. Scale = 10 µm.

**Figure 3:**
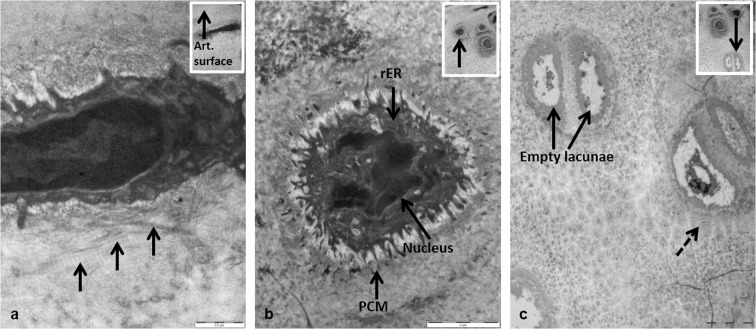
Ultrastructural examination of chondrocytes from different zones of cartilage in a 53 week old BALB/c AKU mouse. (**a**) TEM micrograph of a flattened chondrocyte in the superficial zone of the HAC. Individual collagen fibres, located in the pericellular matrix (PCM), lie parallel to the articular surface (arrowed) (x26,500). Inset: Location of the chondrocyte in HAC (x8250). (**b**) TEM micrograph of a chondrocyte in the deep zone of the HAC. Specific structures within the cell have been labelled (x9900). (**c**) TEM micrograph of hypertrophic chondrons in the ACC. Both sets of chondrocytes appeared chondroptotic with chromatin condensation, cellular disintegration and the final stage of chondroptosis, empty lacunae, all present. Concentric lamellae were also visible surrounding the cells (dashed lines). Inset: Location of the chondrocyte in the HAC (arrowed) (x2500). Inset: Location of the cells in the ACC (x2500). Tissue fixed in glutaraldehyde. Scale = (**a**) 0.5 µm, (**b**) 2 µm, (**c**) 5 µm.

Detailed TEM micrographs from an area of the MFC highlighted the ultrastructure of HAC and ACC, and the cells and collagenous matrices contained within them (Fig. 2a,b). Flattened chondrocytes in the superficial zone lay parallel to the articular surface while chondrocytes in the transitional zone appeared larger and more spherical (Fig. 2a). Higher powered images of chondrocytes in both the superficial and transitional zones showed the presence of collagen fibres in the pericellular matrix (PCM), and increased cellular detail with the nucleus and rough endoplasmic reticulum both visible (Fig. 3a,b). The tidemark, which is the boundary between calcified and non-calcified cartilage, still generates much discussion as to its composition^18–20^. It is highlighted to show the differences between the matrices and cells in non-calcified and calcified articular cartilage (Fig. 2a). Hypertrophic chondrocytes were localised to the ACC (Fig. 2a). Chondroptotic cells, showing chromatin condensation, cellular disintegration and empty lacunae were visible deep in the ACC adjacent to the cement line (Figs 2b & 3c). Surrounding several of these cells we observed the appearance of novel concentric lamellar structures (Figs 2b & 3c, dashed arrows). The lamellae initially looked as if they formed part of the pericellular matrix however upon further examination they could be seen to extend into the territorial matrix.

### Identification of concentric lamellae in the articular calcified cartilage of BALB/c AKU and WT mice

**Figure 4:**
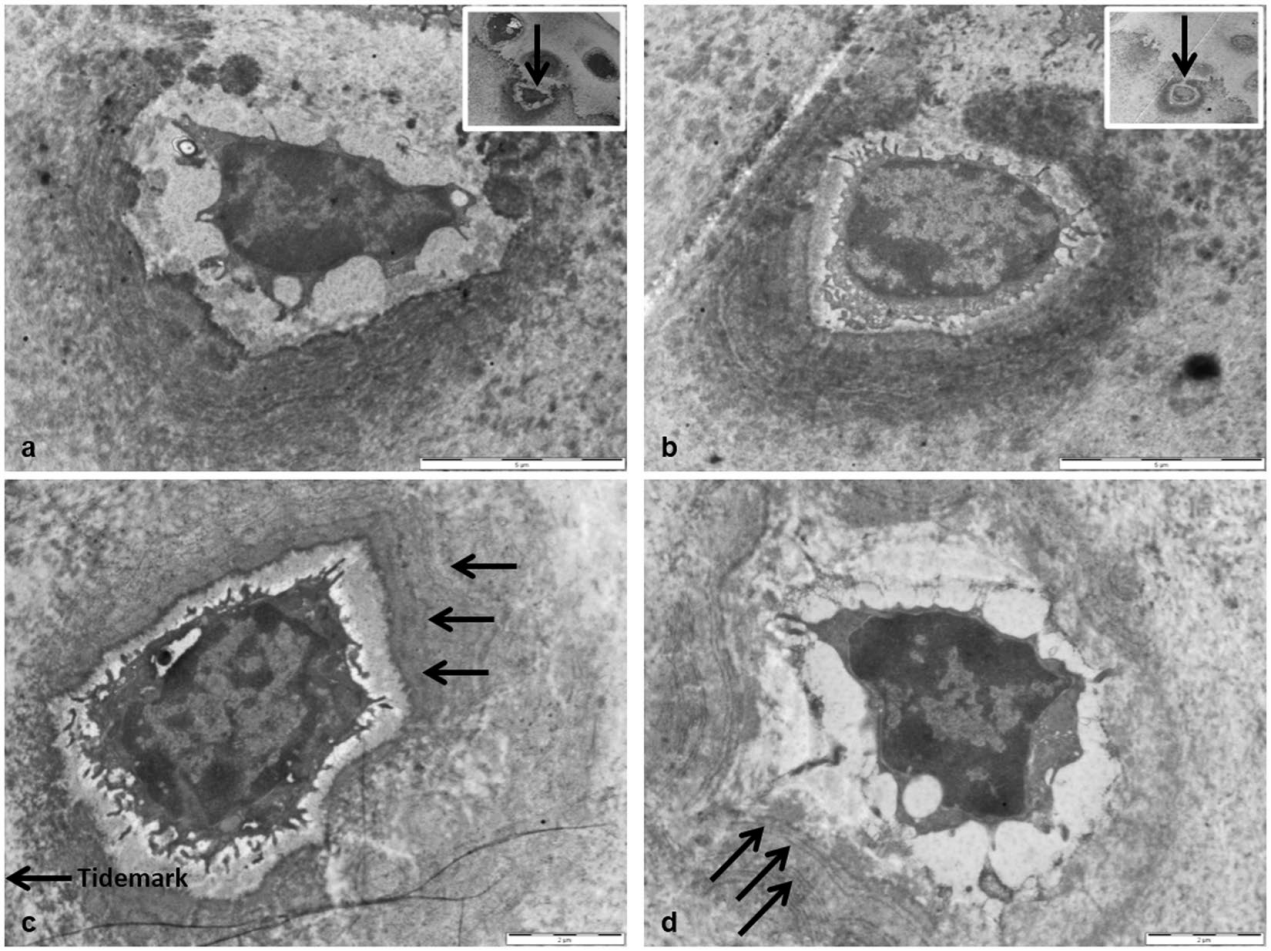
The appearance of concentric lamellae around chondrocytes in the ACC of aged BALB/c AKU mice. (**a**) A chondrocyte partially surrounded by concentric lamella, yet not completely enclosed in the ACC (x6000). Inset: Location of chondrocyte in the ACC, showing apparent ‘opening’ of the tidemark (arrowed) resulting in the cell becoming engulfed by the ACC (x2500). (**b**) A chondrocyte almost completely surrounded by lamellae, progressing deeper into the ACC (x6000). Inset: Location of chondrocyte in the ACC, showing apparent ‘closing’ of the tidemark (arrowed) resulting in the cell becoming completely embedded in the ACC (x2500). (**c**) A chondrocyte surrounded by numerous concentric lamellae (arrowed) in a periodic-like manner (x8200). (**d**) Concentric lamellae surrounding a chondrocyte deep in the ACC, in a periodic manner (arrowed) identical to what was seen in (**c**) (x8200). Tissues fixed in (**a**,**b**) PBFS, (**c**,**d**) glutaraldehyde. Ages = (**a**,**b**) 60 wks, (**c**,**d**) 54.4 wks. Scale = (**a**,**b**) 5 µm, (**c**,**d**) 2 µm.

**Figure 5:**
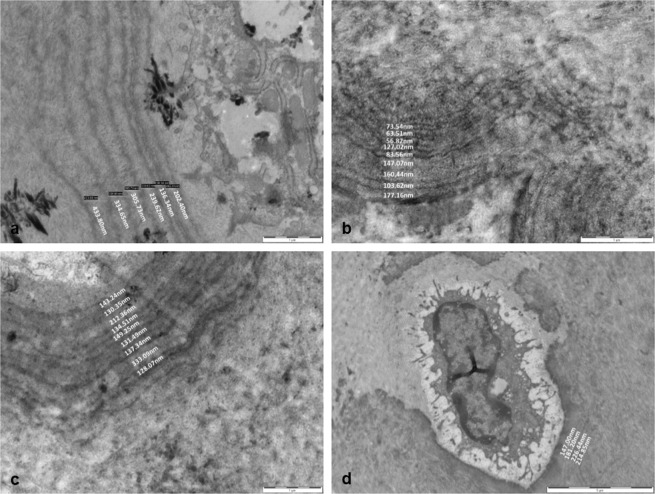
Measurements of concentric lamellae in BALB/c AKU and WT mice. (**a**) Quantification of the lamella in a 7.8 week old AKU mouse showed a general increase in width as they progressed further away from the chondrocyte (x16,500). (**b**,**c**) The number of lamellae surrounding chondrocytes in aged AKU mice (53 + 61 weeks old respectively) increased in comparison to young AKU mice (**a**), however the widths of the lamellae were significantly narrower (x26,500). (**d**) Quantification of the lamellae in an aged WT mouse (69 weeks) revealed the number of lamellae was comparable to that seen in young AKU mice (**a**), whilst the width was comparable to that seen in aged AKU mice (**b**,**c**) (x4200). Tissues fixed in (**a**,**b**) glutaraldehyde (**c**,**d**) PBFS. Scale = (**a**–**c**) 1 µm, (**d**) 5 µm.

As described above our analysis of the cartilage in BALB/c Hgd-/- and WT mice led to the identification of distinct patterns of concentric circles, which we have termed concentric lamellae, surrounding chondrocytes in the ACC. The lamellae were visible both around viable cells, located towards the mineralisation front (Fig. 4a–c) and around hypertrophic and chondroptotic cells located deeper in the ACC, close to the boundary with the SCB (Fig. 4d). Chondrocytes located adjacent to the mineralisation front appeared to be partially engulfed by the lamellae before progressing deeper into the ACC and becoming completely surrounded (Fig. 4a,b). This process appeared to show an apparent opening and closing of the tidemark as the cells became surrounded and embedded in the ACC. The lamellae appeared to be laid down around the chondrocytes in a periodic-like manner (Fig. 4c,d). Chondrocytes located deeper in the ACC had more defined lamellae which enabled us to quantify the lamellae and determine if they became more or less frequent with age, and whether their size was affected by the age of the mice. Eleven samples were subjected to quantitative analysis, three from mice aged 9 weeks and younger, including two AKU and one WT, and a further eight from mice aged 53 weeks and older, including seven AKU and one WT. It was clear from the images that the lamellae found in young AKU mice were fewer in number and thicker in width (Fig. 5a) than in aged AKU mice where they were more frequent but narrower (Fig. 5b,c). Along with more lamellae being present in aged AKU mice there were also more cells affected than in young mice. Although the lamellae were visible in WT mice (Fig. 5d) there did appear to be fewer affected chondrocytes. While the number of lamellae surrounding chondrocytes also appeared to decrease in WT mice, the width remained consistent to those seen in AKU mice of similar age (Fig. 5b,c). This appears to confirm that an increase in the age of the mice leads to a decrease in the width of the lamellae present in the cartilage.

**Figure 6:**
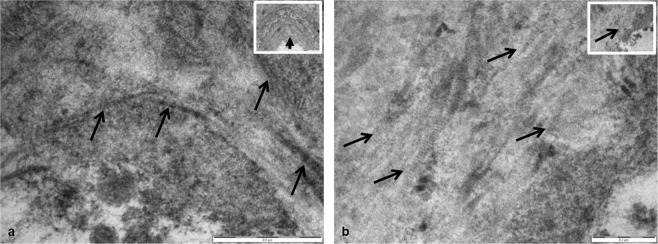
Identification of collagen fibres in aged BALB/c AKU mice. (**a**) Collagen fibres were identified in the lamellae of a 56 week old AKU mouse (arrowed). Periodic banding can be seen along the fibres which is distinctive of collagen (x60,000). Inset: Low power image highlighting the location of the collagen fibres in the lamellae (x16,500). Tissue fixed in glutaraldehyde. (**b**) Collagen fibres were identified in the lamella of a chondron deep in the ACC of a 60 week old AKU mouse. Again, periodic banding can be seen along the fibres which is distinctive of collagen (x87,000). Inset: Low power image highlighting the location of the collagen fibres in the lamella (x43,000). Tissue fixed in PBFS. Scale = (**a**) 0.5 µm, (**b**) 0.2 µm.

Once the lamellae had been identified and quantified we wanted to identify their composition. In a large number of the images it was difficult to ascertain what the lamellae were composed of. However, on further inspection at higher magnification we were able to identify the presence of collagen fibres in the lamellae on a number of the aged AKU mice (Fig. 6a,b). It is clear from both images that collagen fibres were located in the lamellae, particularly those which were located closer to the cell.

## Discussion

TEM was used to detail the ultrastructure of the HAC and ACC of BALB/c AKU mice. These mice are a model of experimental OA due to the osteoarthritic phenotype they show including cartilage degeneration, SCB remodelling and increasing amounts of calcification. The OA phenotype associated with AKU mice provided an opportunity to use TEM to identify any ultrastructural changes in cartilage between AKU and WT mice with the aim of further understanding the role played, particularly that of ACC, in the initiation and progression of OA.

Initial histological examination of AKU mice revealed the presence of concentric ring like structures around chondrocytes deep in ACC at both 31 and 60 weeks of age. Using LM it was not possible to determine what these structures were or to gain any detailed knowledge of their ultrastructure. Further analysis of AKU and WT with TEM also revealed the presence of these concentric ring structures and led to a more robust analysis of the cartilage to try and determine the nature of these structures. Initial signs of typical OA including remodelling of the SCB and protrusion of SCB into ACC were also observed under LM (Fig. 1a,b). Pigmented chondrocytes, a hallmark of AKU, were visible with both H&E and Schmorl’s stain (Fig. 1b,c).

TEM analysis of ACC in AKU mice revealed the presence of concentric lamellae around the majority of chondrocytes scattered throughout this zone of cartilage. The lamellae were also present around chondrocytes in the ACC of WT mice but to a much lesser extent. On initial examination it was unclear if the concentric structures observed under TEM were identical to the ones seen under LM, however the fact they were both present deep in ACC, and not in HAC, suggested possible structural similarities between the two and provided a basis for an in-depth analysis. Extensive literature searches revealed that the presence of these lamellae is a novel finding in the ACC. The structures identified may be related to the lamellae detected using SEM by Hirotani *et al*.^21^, who proposed the existence of a lamellar system around chondrocytes in the deep zone of the articular cartilage in patients with secondary OA. It must be noted however, that these were found only in the HAC and not in the ACC. No definitive reasoning is given by Hirotani *et al*.^21^ for this system of lamellae in the cartilage, although it is suggested it may be as a result of shrinkage from tissue preparation. With no other literature describing this phenomenon, the mechanism behind their formation is not clearly understood. There is evidence, both from the work described in this paper and the results gained by Hirotani that the lamellae are related to the pathogenesis of OA. The lamellae appeared around both viable chondrocytes towards the tidemark, which appeared to be partially engulfed by lamellae, and to a much higher degree around hypertrophic chondrocytes located deep in the ACC (Fig. 4). The fact that they appear much more regularly around hypertrophic chondrocytes may be significant as to the origins of their formation. Hypertrophic chondrocytes are known to express type X collagen^22,23^, and release increased levels of alkaline phosphatase^24^ leading to cartilage calcification^25,26^. Cartilage calcification has been associated with both ageing of tissues^27,28^ and OA pathogenesis^25,29^. Calcification of cartilage associated with OA pathogenesis leads to thinning of HAC^30^ and thickening of ACC^31^, and can be identified by advancement and duplication of the tidemark^32,33^ as mineralisation progresses towards the surface. Thinning of the ACC can also occur during OA if the rate of subchondral remodelling is quicker than the rate of tidemark advancement^34^. The lamellae identified in the ACC appeared to be laid down in association with the advancing tidemark, which would indicate they may be formed during cartilage calcification. Viable chondrocytes at the mineralisation front could be seen to be partially surrounded by the lamellae (Fig. 4a,b). This suggests that chondrocytes in the HAC, which are close to the tidemark, are surrounded by ACC and the lamellae are then laid down during calcification of the cartilage.

The lamellae were identified in both young and aged AKU and WT mice. The greater abundance of lamellae in AKU mice, which are a model of OA, suggests that they may have a role in the pathogenesis of OA. Lamellae were present in young AKU mice (Fig. 5a) suggesting that cartilage calcification and OA initiation begins at a young age in OA mice. There were fewer individual lamellae surrounding the chondrocytes in young AKU mice and they appeared thicker than those seen in aged AKU mice. As the mice increased in age the lamellae became thinner and more frequent around the chondrocytes. This correlates with the increased calcification and cartilage thinning seen in aged mice. Increased calcification which is associated with OA progression^6^, appears to be linked to increasing amounts of lamellae formation around chondrocytes in ACC of aged AKU mice.

Although it is possible the lamellae may be involved in the development and progression of OA, it cannot be discounted that they may be linked to the ageing process. Lamellae were present in both young and aged AKU mice; the number of lamellae around chondrocytes increased in aged AKU mice (Fig. 5). The lamellae were also identified in young and aged WT mice which showed very little cartilage degeneration, suggesting that their formation may have been as a result of the ageing process. Increasing the number of mice examined, over a wide range of ages, should help determine whether the lamellae are linked to either the development of OA or the process of ageing.

Analysis of both AKU and WT mice revealed the appearance of novel concentric lamellae-like structures surrounding hypertrophic chondrons in the ACC. Their possible association with mineralisation and advancement of the tidemark, and their greater abundance in AKU mice indicate that the formation of these lamellae may be involved in the pathogenesis of OA, since thinning of articular cartilage due to advancing mineralisation is reported to be a characteristic of joints undergoing OA. Further work identifying the underlying mechanism(s) by which the lamellae are formed, including immunohistochemistry and Energy Dispersive Spectroscopy (EDAX), should provide a better understanding of the function and regulation of the ACC, and the role of the lamellae in the initiation and progression of OA.
